# Temporal trends in the referral pattern to and yield of cardiac MRI: an analysis from Saudia Arabia

**DOI:** 10.1186/1532-429X-17-S1-P136

**Published:** 2015-02-03

**Authors:** Mouaz H Al-Mallah, Ebtisam Anazi, Ahmed AlJizeeri, Mohsen AlHarthi, Ahmed AlSaileek

**Affiliations:** Cardiac Imaging, King AbdulAziz Cardiac Center, Riyadh, Saudi Arabia

## Background

Cardiac MRI was recently introduced in Saudia Arabia and is only available in few centers. The aim of this analysis is to describe the current referral pattern to cardiac MRI in Saudia Arabia.

## Methods

This is a single center study that included patients who were referred for cardiac MRI for evaluation of clinical indications. The indications and baseline demographics of patients referred to cardiac MRI were collected. The different indications were compared in each year.

## Results

A total of 1,581 patients (38% females) were included. In first few years of the initiation of the service, most patients were referred for assessment of viability (54%) followed by assessment of etiology of cardiomyopathy (19%). However, in the subsequent years, viability referrals decreased and there was a steady increase in other indications (assessment of LV function, arrhythmias, cardiac masses, congenital heart disease,..etc.) (figure). Referral to stress MRI continued to be very low (less than 1%). 46% of the reoffered patients had an ejection fraction less than 50% and 42% of the patients had evidence of myocardial delayed enhancement.

**Figure 1:**
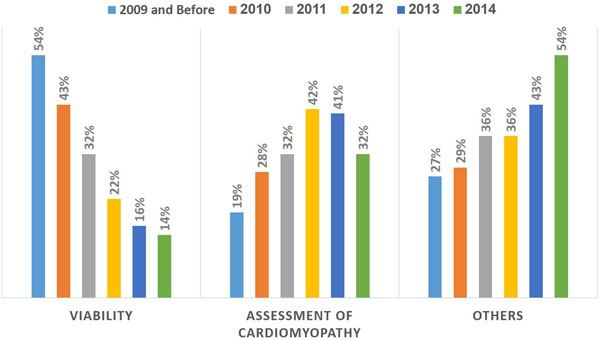
Figure 1

## Conclusions

In the first years of establishing a cardiac MRI program, assessment of viability and cardiomyopathy constitute more than two thirds of referral to cardiac MRI.

## Funding

None.

